# Supplementary material for: Working strokes produced by curling protofilaments at disassembling microtubule tips can be biochemically tuned and vary with species
Source: eLife. 2022 Dec 29;11:e83225. doi: 10.7554/eLife.83225 (PMC9799970; doi:10.7554/eLife.83225)
Supplement: Figure 4—source data 2. — Each row is colored to reflect the method used to estimate protofilament curvature. [file elife-83225-fig4-data2.docx]

**Figure 4–source data 2: Table of estimates of protofilament curvature reported in the literature.** Each row is colored to reflect the method used to estimate protofilament curvature.

| ***Curvature (degrees per dimer)*** | ***SE*** | ***Meaured from*** | ***Ligand(s)*** | ***Description*** | ***Reference*** |
| --- | --- | --- | --- | --- | --- |
| 27.8 |  | Cryo 2D | Presumed GDP | Curvature measured from diameter of PF curl 1 mM Mg2+ | Mandelkow 1991 |
| 24.8 |  | Cryo 2D | Presumed GDP | Curvature measured from diameter of PF curl 20 mM Mg2+ | Mandelkow 1991 |
| 20.2 | 12.1 | Cryo-ET trace | Presumed GDP | Cytoskeleton porcine tubulin grown from axonemes, with disassembly induced by isothermal dilution, BRB80 (1 mM Mg^2+^). Average length of 43 ± 16 nm | McIntosh 2018 |
| 19.3 | 13.1 | Cryo-ET trace | Presumed GTP | Cytoskeleton porcine tubulin grown from axonemes, BRB80 (1 mM Mg^2+^). Average length of 36 ± 15 nm | McIntosh 2018 |
| 26 | N/A | crystal structure | stathmin, GDP, colchicine | Colchicine known to induce curvature, curvature looks very close by eye when aligned by α-tub to structures submitted by Nawrotek et al. | Ravelli 2004 |
| 22 | N/A | crystal structure | DARPin, GTP | DARPin binds at exposed terminus of β-tub, structure shows GTP bound at both E and N-sites. | Pecquer 2012 |
| 19 | N/A | crystal structure | stathmin, GTP, GDP, GmpCpp | regardless of ligand at E-site, curvature remains seemingly constant. Stathmin may impose substantial influence on curvature* | Nawrotek 2011 |
| 21 | 13 | Cryo-ET trace | Presumed GTP | 10 µM Cytoskeleton porcine tubulin, growing MT | Gudimchuck 2020 |
| 21 | 14 | Cryo-ET trace | Presumed GTP | 20 µM Cytoskeleton porcine tubulin, growing MT | Gudimchuck 2020 |
| 18 | 13 | Cryo-ET trace | Presumed GTP | 40 µM Cytoskeleton tubulin, growing MT | Gudimchuck 2020 |
| 21 | 11 | Cryo-ET trace | Presumed GTP | α1B/βI+βIVb tubulin, growing | Gudimchuck 2020 |
| 22.9 |  |  |  | Assumed curvature of relaxed tubulin per model proposed (conversion from 0.2 rad) | Gudimchuck 2020 |
| 12 | 12.3 | Cryo-ET trace | In vivo | KMT-PTK-meta 51 ± 21 nm length, 1,465 PF traces | McIntosh 2018 |
| 13.7 | 11.8 | Cryo-ET trace | In vivo | KMT-PTK-ana 13.7 ± 11.8 nm length, 192 PF traces | McIntosh 2018 |
| 17.8 | 12.8 | Cryo-ET trace | In vivo | KMT-Chlamy-meta 42 ± 15 nm length, 700 PF traces | McIntosh 2018 |
| 16.3 | 13.6 | Cryo-ET trace | In vivo | KMT-Chlamy-ana 54 ± 14 nm length, 452 PF traces | McIntosh 2018 |
| 17.9 | 14.5 | Cryo-ET trace | In vivo | KMT-CEleg-meta 44 ± 17 nm length, 315 PF traces | McIntosh 2018 |
| 22.9 | 19.1 | Cryo-ET trace | In vivo | KMT-CEleg-ana 49 ± 18 nm length, 461 PF traces | McIntosh 2018 |
| 21.1 | 17.5 | Cryo-ET trace | In vivo | KMT-Cerevis-meta 27 ± 11 nm length, 449 PF traces | McIntosh 2018 |
| 24.6 | 81.9 | Cryo-ET trace | In vivo | KMT-Cerevis-ana 31 ± 12 nm length, 539 PF traces | McIntosh 2018 |
| 11.5 | 5.7 | Oligomer fit (Cryo 2D) | GTP | Oligomers in early stages of assembly (Drosophila tubulin), GTP | Ayukawa 2021 |
| 15.8 | 5.6 | Oligomer fit (Cryo 2D) | GDP | Oligomers in early stages of assembly (Drosophila tubulin), GDP | Ayukawa 2021 |
| 9.4 |  | Cryo-ET |  | Tubulin sheets at MT tips (density is indistinguishable from dark moire fringe that defines MT wall, indicating multiple PF's in gently curving sheet. | Guesdon 2016 |
| 1 |  | Cryo 2D |  | Finds miniscule curvatures from MT groups/sheets nucleated from axonemes (~1 deg/dimer) | Orbach and Howard 2019 |
| 25.8 | 0.21 | Cryo 2D | GTP or GDP | measured by ring diameter of curling PF's at tips, did not differentiate between assembling and disassembling | Vemu 2017 |
| 8 |  | Oligomer trace | GTP | 50 µM brain tub | Howes 2018 |
| 10 |  | Oligomer trace | GDP | 50 µM brain tub | Howes 2018 |
| 6 |  | Oligomer trace | GTP | 10 µM yeast tubulin | Howes 2018 |
| 8 |  | Oligomer trace | GDP | 10 µM yeast tubulin | Howes 2018 |
| 23.5 | 6 | Cryo 2D | GDP, 4 mM Ca2+ | bovine brain tubulin | Muller-Reichart 1998 |
| 22.3 | 6 | Cryo 2D | GDP, 40 mM Ca2+ | bovine brain tubulin | Muller-Reichart 1998 |
| 12.2 | 2.8 | Cryo 2D | GmpCpp, Ca2+ | bovine brain tubulin | Muller-Reichart 1998 |
