# Supplementary figures and images for: Working strokes produced by curling protofilaments at disassembling microtubule tips can be biochemically tuned and vary with species
Source: eLife. 2022 Dec 29;11:e83225. doi: 10.7554/eLife.83225 (PMC9799970; doi:10.7554/eLife.83225)

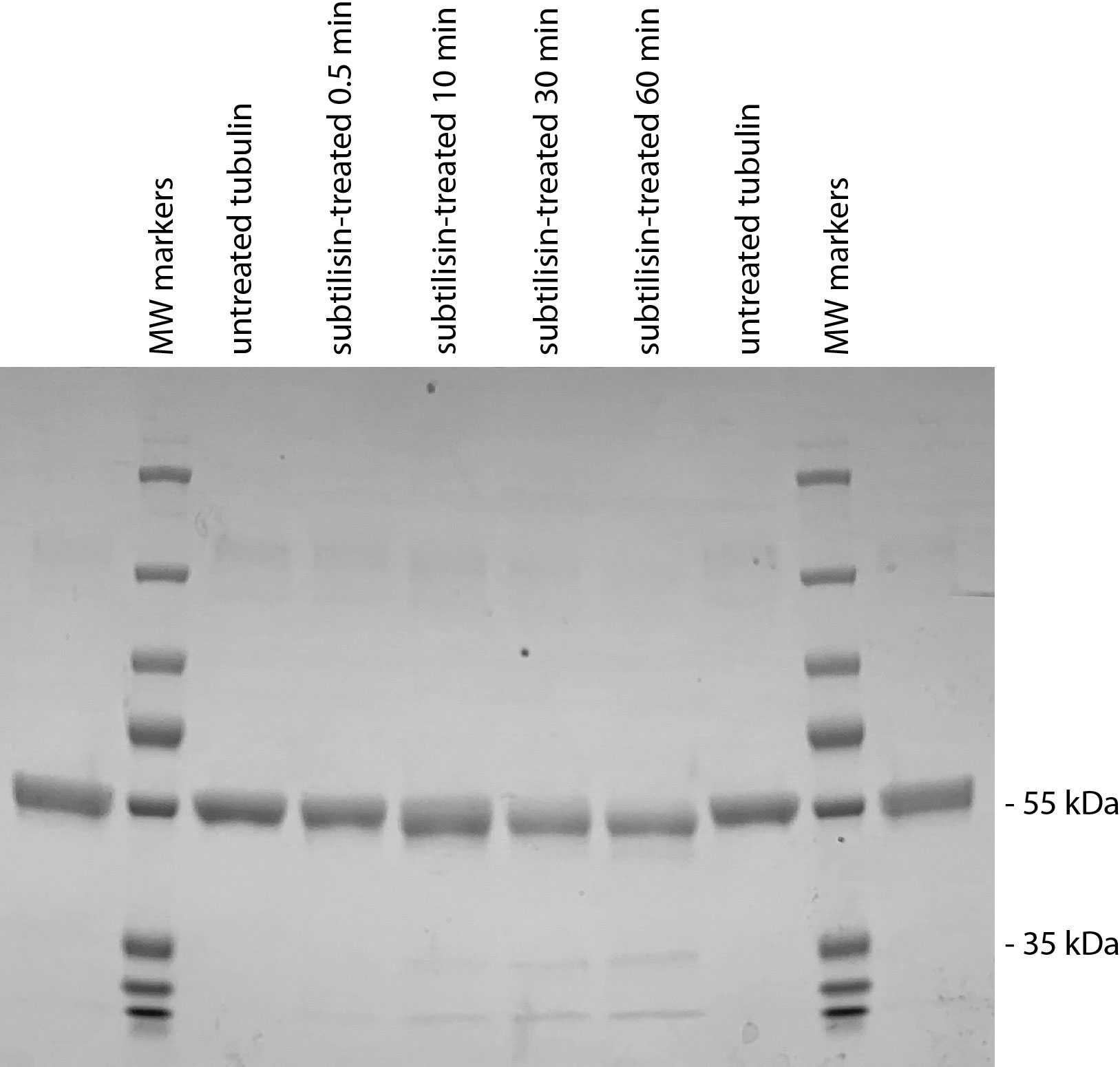

Supplement: Figure 5—source data 1. — This image is provided as a JPG file with relevant lanes labeled. [file elife-83225-fig5-data1.zip › Figure5_Source_Data1_Coomassie.jpg]

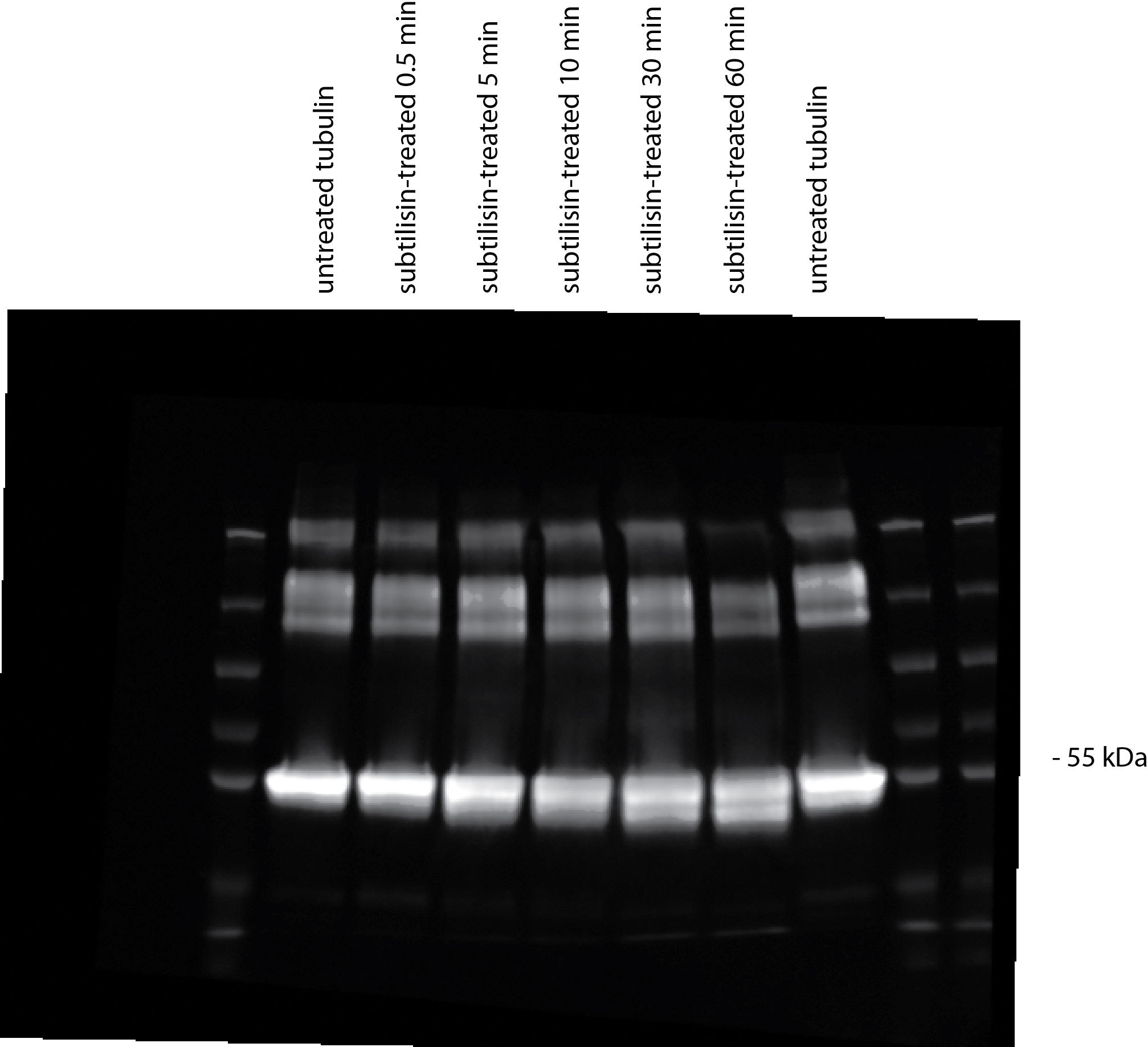

Supplement: Figure 5—source data 2. — This image is provided as a JPG file with relevant lanes labeled. [file elife-83225-fig5-data2.zip › Figure5_Source_Data2_antiAlpha.jpg]

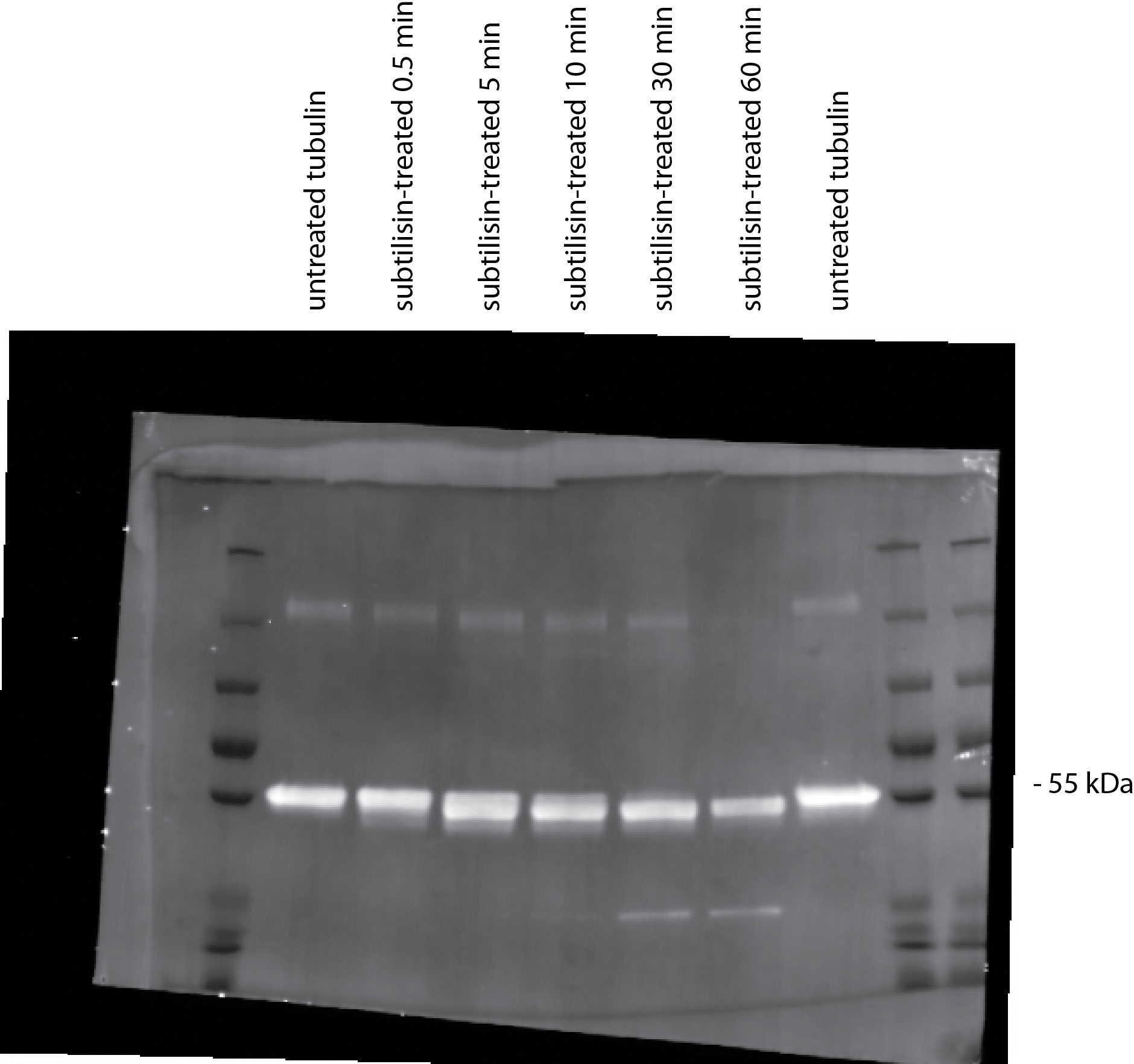

Supplement: Figure 5—source data 3. — This image is provided as a JPG file with relevant lanes labeled. [file elife-83225-fig5-data3.zip › Figure5_Source_Data3_antiBeta.jpg]

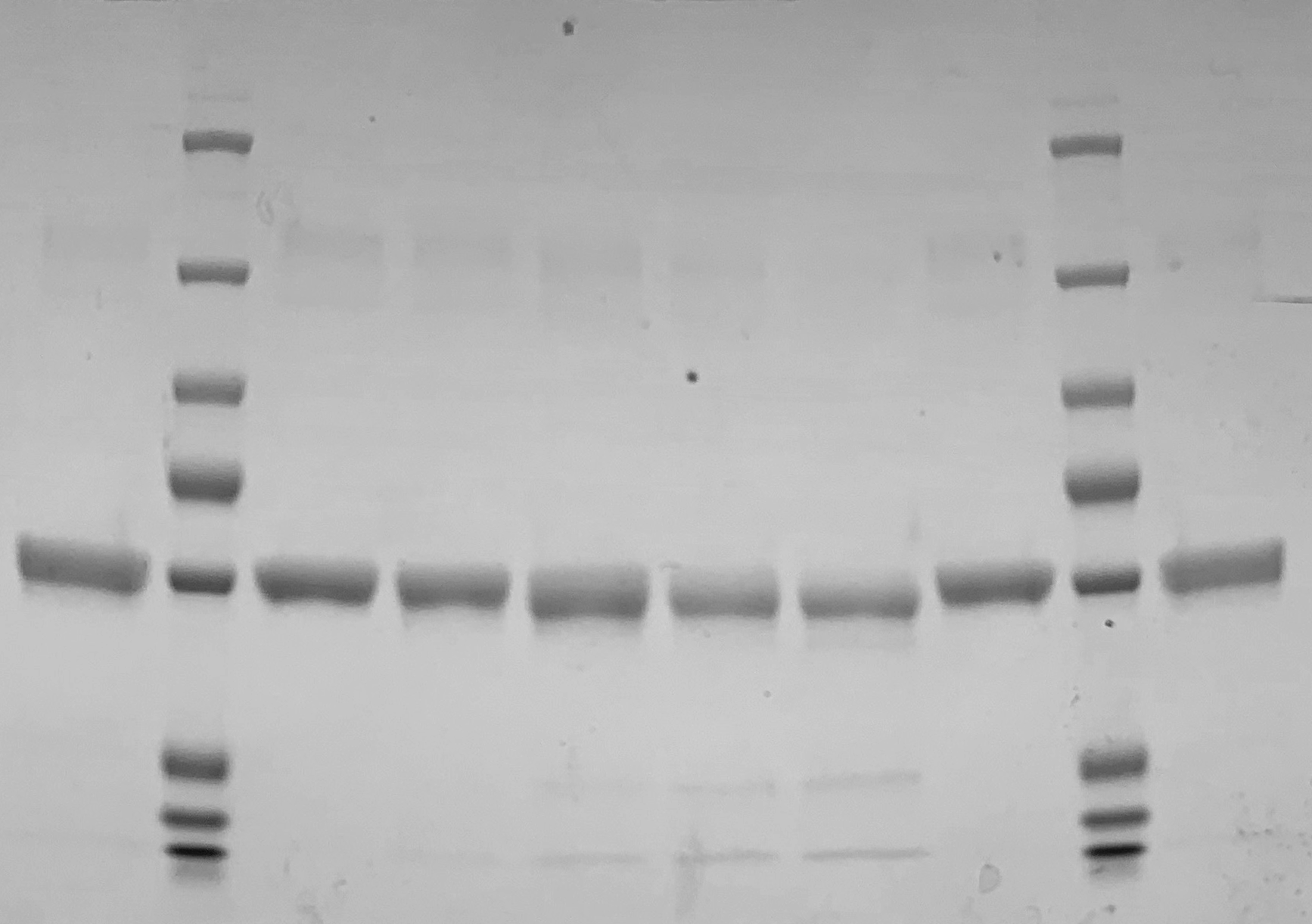

Supplement: Figure 5—source data 5. — This full raw unedited image is provided as a PNG file. [file elife-83225-fig5-data5.zip › Figure5_Source_Data5_RAW_coomassie.png]

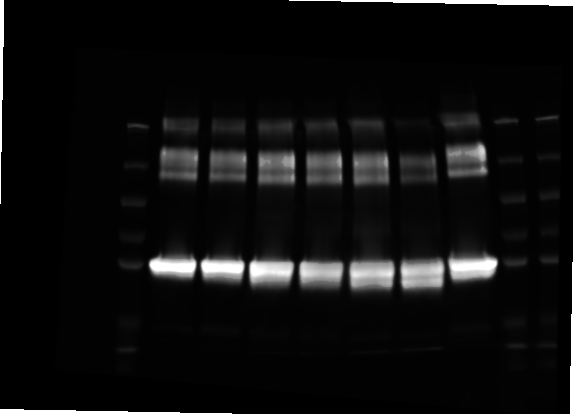

Supplement: Figure 5—source data 6. — This full raw unedited image is provided as a TIF file. [file elife-83225-fig5-data6.zip › Figure5_Source_Data6_RAW_antiAlpha.tif]

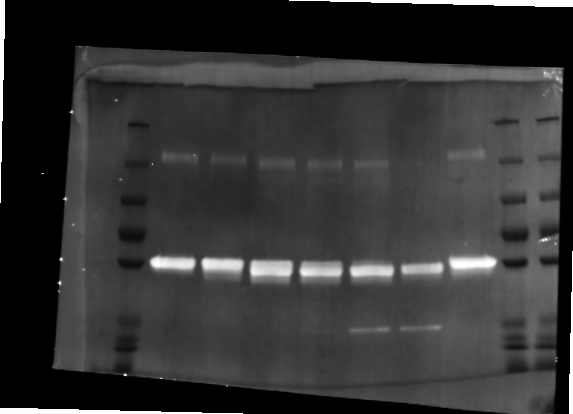

Supplement: Figure 5—source data 7. — This full raw unedited image is provided as a TIF file. [file elife-83225-fig5-data7.zip › Figure5_Source_Data7_RAW_antiBeta.tif]
